# Cordycepin Regulates GSK-3β/β-Catenin Signaling in Human Leukemia Cells

**DOI:** 10.1371/journal.pone.0076320

**Published:** 2013-09-26

**Authors:** Bor-Sheng Ko, Yi-Jhu Lu, Wen-Ling Yao, Tzu-An Liu, Shean-Shong Tzean, Tang-Long Shen, Jun-Yang Liou

**Affiliations:** 1 Institute of Cellular and System Medicine, National Health Research Institutes, Zhunan, Taiwan; 2 Department of Internal Medicine, National Taiwan University Hospital, Taipei, Taiwan; 3 Department of Plant Pathology and Microbiology, National Taiwan University, Taipei, Taiwan; 4 Graduate Institute of Basic Medical Science, China Medical University, Taichung, Taiwan; Winship Cancer Institute of Emory University, United States of America

## Abstract

**Background:**

Leukemia stem cells (LSCs) are a limitless cell source for the initiation and maintenance of leukemia. Activation of the Wnt/β-catenin pathway is required for the survival and development of LSCs. Therefore, targeting β-catenin is considered a therapeutic strategy for the treatment of leukemia. The goal of this study was to explore whether cordycepin, an active component of the traditional medicine *Cordyceps sinensis*, regulates β-catenin expression in leukemia cells.

**Methodology and Principal Findings:**

In this study, we found that cordycepin significantly suppressed cell proliferation in all malignant cancer cells, including U937, K562, A549, HepG2, SK-Hep1 and MCF7 in a dose-dependent manner. However, cordycepin reduced β-catenin levels in U937, K562 and THP1 leukemia cells and had no effect on other solid cancer cells. In addition, treatment with cordycepin significantly suppressed leukemia colony formation in soft agar assay. Cordycepin enhanced proteasome-dependent degradation and inhibited nuclear translocation of β-catenin in leukemia cells. Cordycepin-reduced β-catenin stability was restored by the addition of a pharmacological inhibitor of GSK-3β, indicating that cordycepin-suppressed β-catenin stability is mediated by the activation of GSK-3β. Furthermore, cordycepin abolished the effect of Wnt3a-induced β-catenin in leukemia cells. In addition, cordycepin-impaired β-catenin is regulated by Akt activation but is not significantly influenced by AMPK or mTOR signal pathways.

**Significance:**

Our findings show for the first time that codycepin selectively reduces β-catenin stability in leukemia but not in other solid tumor cells. This suppressive effect is mediated by regulating GSK-3β. A synergistic combination of cordycepin with other treatments should be used as a novel strategy to eradicate leukemia *via* elimination of LSCs.

## Introduction

β-catenin, the main downstream effector of the canonical Wnt pathway, is implicated in governing self-renewal of various normal and cancer stem cells [1]–[3]. At the basal state, β-catenin stability is controlled by a complex composed of multiple proteins including: axin, casein kinase (CK), adenomatous polyposis coli (APC) and glycogen synthase kinase 3β (GSK-3β) [4]–[6]. Complex-associated β-catenin is phosphorylated by GSK-3β and consequently degraded *via* the proteasome-dependent pathway [4]–[6]. Upon Wnt activation which subsequently disrupts and inactivates GSK-3β, β-catenin disassociates from the complex, resulting in increased nuclear translocation where β-catenin regulates the expression of renewal and proliferation genes [5], [6]. In human leukemia, Wnt/β-catenin signaling contributes to the development of leukemia stem cells (LSCs) in both acute myeloid leukemia (AML) and chronic myeloid leukemia (CML) [7]–[11]. Expression of β-catenin in AML predicts enhanced clonogenic capacities and associates with poor prognosis [12]. Moreover, β-catenin is involved in maintaining the survival of LSCs that are insensitive to kinase inhibition in mice with BCR-ABL-induced CML [9]. In addition, impairment of Wnt/β-catenin signaling synergizes with imatinib to delay CML disease recurrence [13]. Thus, abrogation of β-catenin signaling is a potential strategy for treating leukemia *via* LSCs eradication.

One of the commonly used traditional Chinese medicine, Dong Chong Xia Cao is comprised of the complex of the fungus *Cordyceps sinesis* and its infected larvae, *Hepialus armoricamus* [14], [15]. This complex has long been used as a health food, and its high potency in treating various diseases has been extensively reported. The extracts of Dong Chong Xia Cao have been frequently documented as immune activators, anti-aging and anti-tumor effectors [14], [15]. Cordycepin, also now known as 3-deoxyadenosine, is a major active ingredient in the extracts of Dong Chong Xia Cao. As an adenosine analogue, cordycepin suppresses the activities of polyadenylate polymerase (PAP) and terminates mRNA synthesis prematurely which results in cell death. Furthermore, cordycepin was reported to induce apoptosis in various types of cancer cells. For example, cordycepin was shown to induce antitumor effect or cell apoptosis in human head-and-neck squamous cell carcinoma cells [16], bladder cancer cells [17], thyroid carcinoma cells [18], breast cancer cells [19], multiple myeloma cells [20], leukemia [21]–[23], lymphoma cells [24] and mouse leydig tumor cells [25]. In addition, the inhibitory effect of cordycepin was demonstrated on hematogenic metastasis of mouse melanoma cells [26], [27] and lung carcinoma cells [28]. Cordycepin was shown to promote cell cycle arrest by regulating c-Jun N-terminal kinase in human bladder and colon cancer cells [29], [30]. In hematological malignancies, cordycepin has cytotoxic and apoptogenic effects via the inactivation of PAP and the subsequent inhibition of mRNA polyadenylation [24]. These effects are more prominent in terminal deoxynucleotidyl transferase-positive leukemic cells [24]. In addition, other cytotoxic or protective agents such as hydroxyurea or deoxycorfomycin have been shown to enhance the anti-tumor effects of cordycepin [31], [32].

Although cordycepin was shown to have anti-leukemia properties [21]–[23], to our knowledge the molecular mechanisms of cordycepin involved in suppressing leukemia development have never been elucidated. In this study, we show that cordycepin selectively suppresses cell proliferation *via* regulating GSK-3β/β-catenin signaling in leukemia cells. Our results suggest that cordycepin can synergize with other anti-leukemia reagents by targeting LSCs to treat leukemia.

## Materials and Methods

### Cell Culture and Reagents

U937, K562 and THP1 cells were maintained in RPMI 1640 medium (Gibco, Gaithersburg, MD) and A549, HepG2, SK-HEP-1 and MCF-7 cells were maintained in Dulbecco’s modified Eagle’s medium (DMEM) (Gibco, Gaithersburg, MD) supplemented with 10% fetal bovine serum (Biological Industries, Kibbutz Beit-Haemek, Israel), 100 units/ml penicillin, 100 µg/ml streptomycin (Gibco, Gaithersburg, MD) at 37°C in a 5% CO_2_ humidified incubator. Cordycepin, adenosine, SB216763 and rapamycin were purchased from Sigma-Aldrich (St. Louis, MO). Compound c and MG-132 were from Merck KGaA, (Darmstadt, Germany). Ly-294002 was obtained from Enzo Life Sciences (New York City, NY) and Wnt-3a was from R&D Systems (Minneapolis, MN).

### Cell Proliferation Assay

Cell proliferation of adherent cells (A549, HepG2, and SK-Hep1) was determined using a 3-(4,5-dimethylthiazol-2-yl)-2,5-diphenyltetrazolium bromide (MTT) assay described previously [33]. Briefly, cells were plated for 24 and 48 hrs and subsequently treated with cordycepin or other indicated reagents and absorbance measured at 570 nm. U937 and K562 cells were plated for 24 and 48 hrs and subsequently treated with indicated reagents. Cell proliferation was measured by using the Cell-Titer 96® AQueous One Solution Cell Proliferation MTS (3-(4,5-dimethylthiazol-2-yl)-5-(3-carboxymethoxyphenyl)-2-(4-sulfophenyl)-2H-tetrazolium) Assay kit (Promega, Madison, WI) [34], [35]. Absorbance was measured at 490 nm using an ELISA plate reader.

### Western Blot Analysis

Protein expression was determined by Western blot analysis described previously [36]. Cells treated with indicated reagents were harvested and lysed using ice cold RIPA buffer (0.5 mol/L Tris-HCl, pH 7.4, 1.5 mol/L NaCl, 2.5% deoxycholic acid, 10% NP-40, 10 mmol/L EDTA; Millipore, Temecula, CA) containing cocktail protease inhibitors (Roche, Indianapolis, IN). Cell lysates were harvested by centrifugation at 16,100 *g* at 4°C for 20 minutes. 20 µg of proteins from each sample were applied to the gradient SDS-PAGE gel and immunoblotted onto PVDF membranes. The membranes were blocked, incubated with primary antibodies against Akt1, Phospho-Akt (Ser-473), GSK-3β, Phospho-GSK-3β (Ser9) and Cyclin D1 (Cell Signaling Technology, Beverly, MA), β-catenin (BD Biosciences, San Jose, CA), β-actin (Sigma-Aldrich St. Louis, MO) or Lamin A/C (Santa Cruz Biotechnologies, Heidelberg, Germany), followed by an incubation with a secondary antibody conjugated horseradish-peroxidase. Protein levels were determined by the use of enhanced chemiluminescence reagents.

### Immunofluorescent Staining

Immunofluorescence staining was performed as described previously [33], [37]. Briefly, U937 cells were treated with 100 µM cordycepin for 24 h followed by fixed and permeabilized with 2% paraformaldehyde at 4°C for 15 minutes and 0.1% Triton X-100 in PBS for additional 5 minutes. After blocking with PBS containing 10% FBS, cells were incubated with the primary antibodies against anti-β-catenin (BD Biosciences, San Jose, CA), followed by incubation with Alexa Fluor® 488 secondary antibody (Invitrogen, Grand Island, NY) for 2 hours. Samples were mounted and images were analyzed by use of the Leica TCS SP5 Confocal Imaging System (Leica, Germany).

### Colony Forming Assay

A colony forming assay was performed using a soft-agar assay [38], [39]. Briefly, 4,000 U937 cells were suspended and seeded in 2 mL of RPMI 1640 medium containing 2% FBS, cordycepin (0–50 µM) and 1% low-melting agarose (SeaPlaque® Agarose, Lonza Rockland, Inc.). This was followed by an overlay of 1 ml of 0.5% low-melting agarose. After three weeks, the colonies were stained with 0.005% crystal violet in 25% methanol. Colony numbers were counted and representative images were captured.

### Preparation of Nuclear Fractions

U397 cells treated with indicated reagents were harvested and nuclear as well as cytosolic proteins were extracted and prepared by using the ProteoExtract® Subcellular Proteome Extraction Kit (EMD Millipore Corporation, Billerica, MA) as described previously [40]. Proteins levels were determined by Western blot analysis.

### Statistical Analysis

The Student’s *t*-test was used to analyze differences between 2 experimental groups. A *p* value less than 0.05 was considered statistically significant.

## Results

### Cordycepin Suppresses Cancer Cell Proliferation

**Figure 1:**
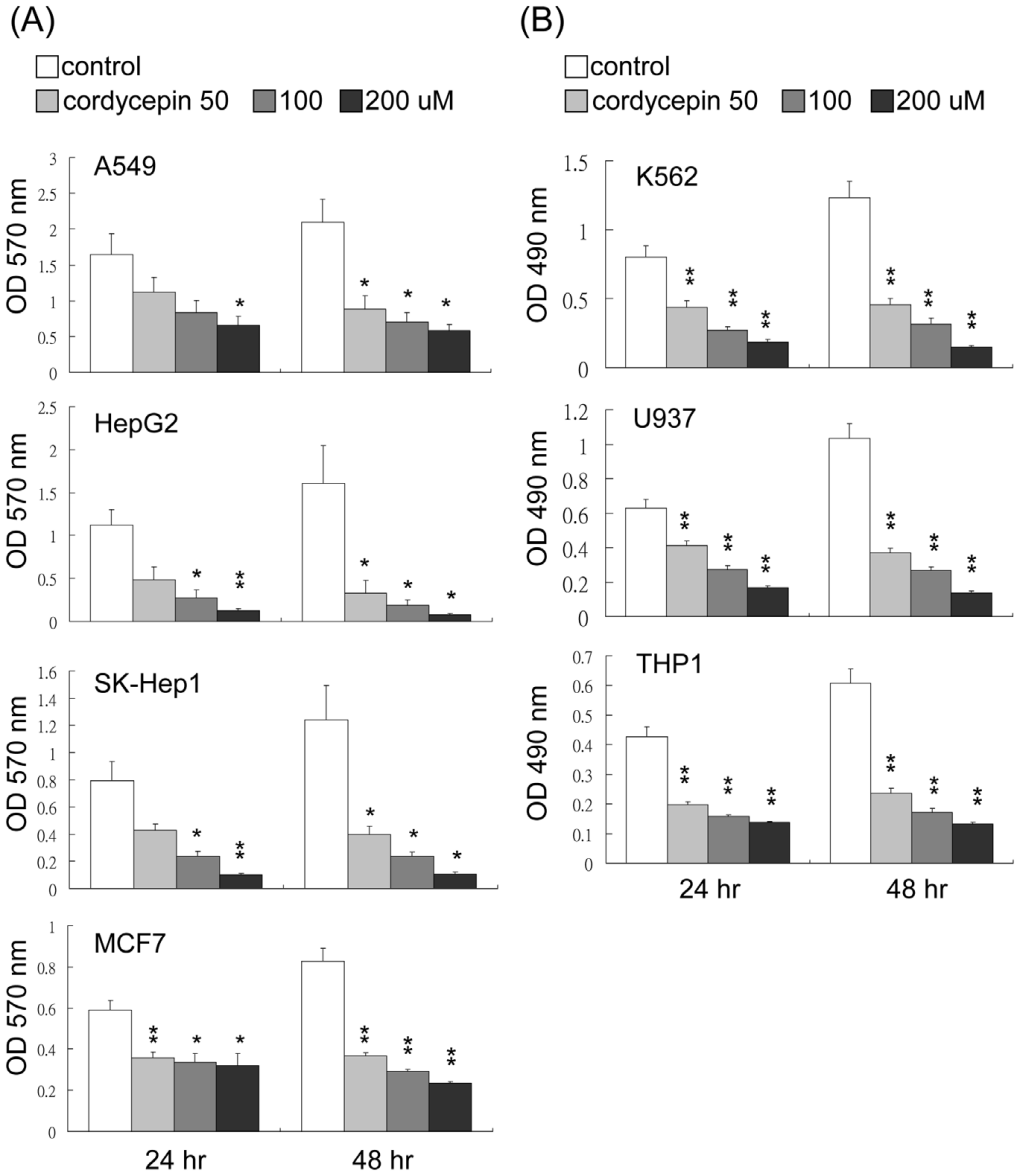
Cordycepin suppresses cell proliferation in various cancer cells. (A) A549, HepG2, SK-Hep1 and MCF7 cells were treated with cordycepin at 50–200 µM for 24 hrs and 48 hrs and cell proliferation was determined by an MTT assay. (B) U937, K562 and THP1 cells were treated with cordycepin at 50–200 µM for 24 hrs and 48 hrs and cell proliferation was determined by an MTS assay. These data are from three independent experiments. Each bar denotes mean ± S.E.M. *, *P*<0.05, **, *P*<0.01.

To explore the potential role of cordycepin in modulating tumor growth, several solid and suspension cancer cells including U937, K562, THP1, A549, HepG2, SK-Hep1 and MCF-7 cells were treated with cordycepin (50 to 200 µM) for 24–72 hrs. Cell proliferation rates were determined by MTS or MTT assays for suspension and adherent cells, respectively. Cordycepin significantly inhibited cell proliferation of all cell lines in a dose- and time-dependent manner (Figure 1A and 1B). We next examined whether this suppressive effect is selectively induced by cordycepin. A549, U937 and K562 cells were treated with different concentrations of adenosine (50 to 200 µM) for 24–72 hrs and cell proliferation assays were determined by MTT and MTS assays. Although adenosine has only a minimal effect on cell proliferation (Figure S1), cordycepin exerted a more profound effect on cell proliferation.

### Cordycepin Reduces β-catenin Expression and Colony Formation in Leukemia Cells

**Figure 2:**
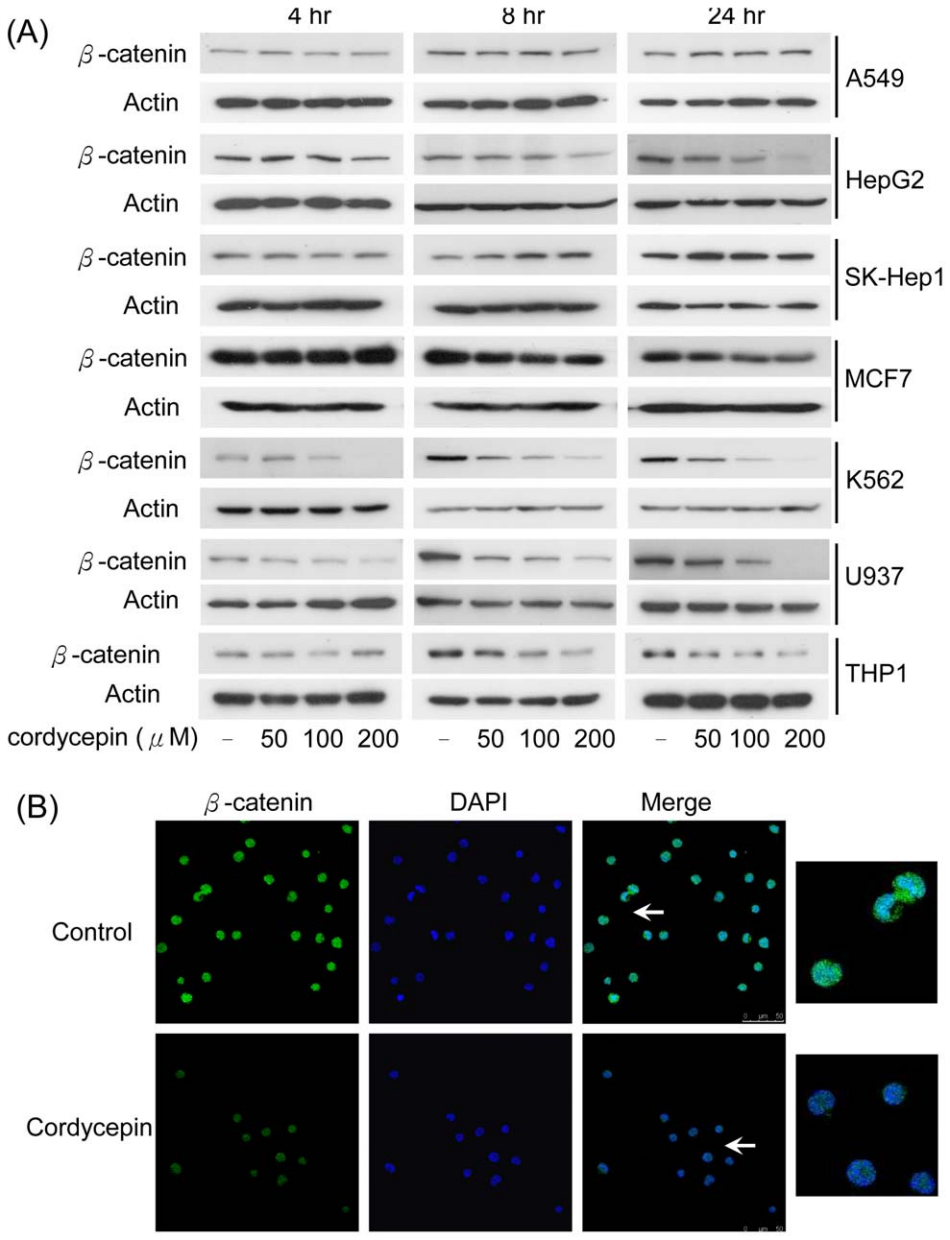
Effect of cordycepin on β-catenin expression. (A) A549, HepG2, SK-Hep1, MCF7, K562, U937 and THP1 cells were treated with different concentrations of cordycepin for 4 to 24 hrs. β-catenin protein levels were determined by Western blot analysis. Actin was used as a loading control. (B) Expression level and subcellular localization of β-catenin was examined by immunofluorescent confocal microscopy. The right panels are magnified images for the indicated area of arrows.

**Figure 3:**
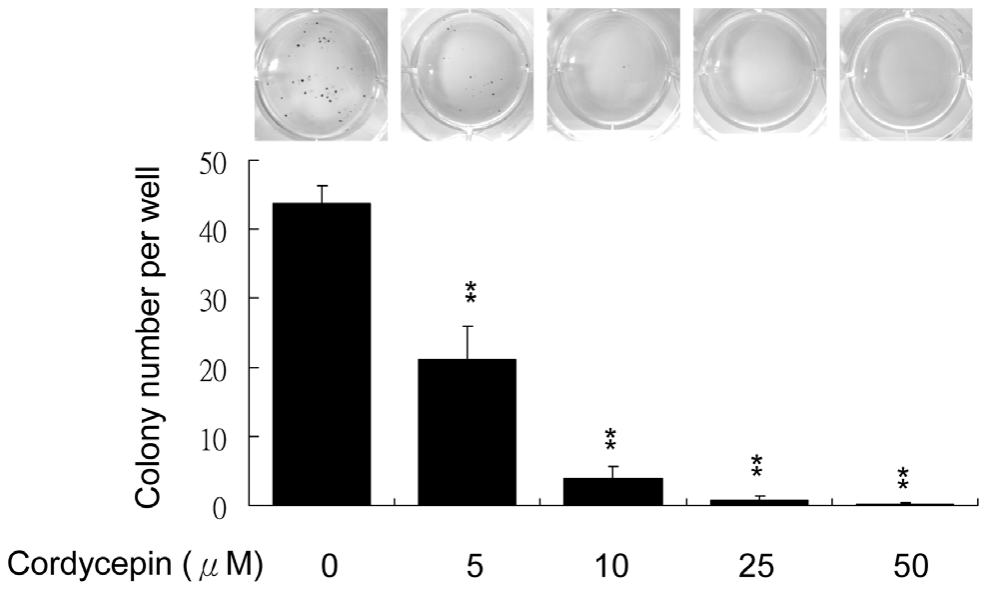
Cordycepin suppresses colony formation of leukemia cells. U937 cells were treated with indicated concentrations (5 to 50 µM) of cordycepin and colony formation was determined by a soft agar assay. These data are from three independent experiments. Each bar denotes mean ± S.E.M. *, *P*<0.01.

β-catenin is considered an important regulator of cell proliferation in various cell types including malignant cancer cells. We examined whether cordycepin affects β-catenin expression. Solid and suspension cancer cells were treated with 50 to 200 µM of cordycepin for 4 to 24 hrs and the expression of β-catenin was determined by Western blotting. Cordycepin dramatically reduced β-catenin expression in a dose-dependent manner in K562, U937 and THP1 cells (Figure 2A). However, treatment of cordycepin had no significant effect on β-catenin in solid cancer cells, except for a slight reduction in HepG2 cells at the higher concentration (Figure 2A). The reduced expression of β-catenin in U937 cells following treatment with cordycepin was confirmed by confocal microscopy (Figure 2B). Both total and nuclear β-catenin level was significantly suppressed by cordycepin (Figure 2B, right panel). In contrast, treatment with various concentrations of adenosine did not affect β-catenin expression in U937, K562 and A549 cells (Figure S2). Furthermore, although cordycepin reduced β-catenin in U937 but not in A549 cells, expression of cyclin D was downregulated in both cells (Figure S3). As β-catenin was suggested to play as a crucial role in maintaining leukemia stem cell survival and renewal [9], we therefore examined the effect of cordycepin on the colony forming capability of leukemia cells. U937 cells were seeding in soft agar containing medium and indicated concentrations of cordycepin (5 to 50 µM). Cordycepin significantly reduced colony forming numbers in a concentration-dependent manner (Figure 3). These results indicate that cordycepin selectively suppresses β-catenin in leukemia cells and may potentially inhibit the survival and renewal of LSCs.

### Cordycepin Suppresses and Inactivates β-catenin *via* the Proteosome-dependent Degradation

**Figure 4:**
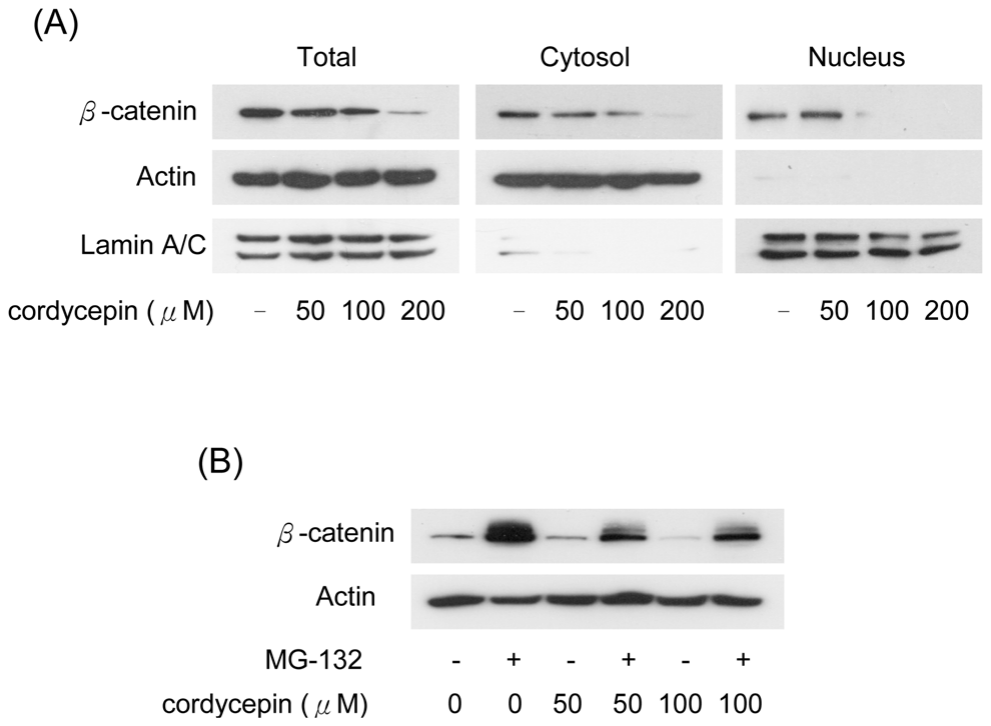
Cordycepin reduces β-catenin via proteasome dependent degradation. (A) U937 cells were treated with different concentrations (50 to 200 µM) of cordycepin. Nuclear and cytosolic fractions were extracted and the expression of β-catenin was determined by Western blot analysis. Actin and lamina/C were used as the loading control for cytosolic and nuclear fractions, respectively. (B) U937 cells were treated with 50 or 100 µM of cordycepin combined with/without MG-132 (10 µM) for 4 hrs. Expression levels of β-catenin were determined by Western blot analysis. Actin was used as a loading control.

The accumulation of β-catenin results in nuclear translocation, thereby inducing the expression of downstream genes. We determined the effect of cordycepin on the nuclear translocation of β-catenin by Western blots in subcellular fractions of U937 cells. β-catenin levels were eliminated in total cell lysates, cytosolic and nuclear fractions in cordycepin-treated U937 cells (Figure 4A). As the protein level of β-catenin is regulated by the proteasome-dependent pathway, we examined whether the suppression of β-catenin was modulated by the reduction of protein stability. U937 cells were treated with cordycepin combined with MG-132, a proteasome inhibitor and the cordycepin-reduced β-catenin levels were significantly restored by MG-132 (Figure 4B). These results indicate that cordycepin suppresses β-catenin by increasing proteasome-dependent degradation of β-catenin in leukemia cells.

### Cordycepin Regulates β-catenin *via* Wnt/GSK-3β Signaling

**Figure 5:**
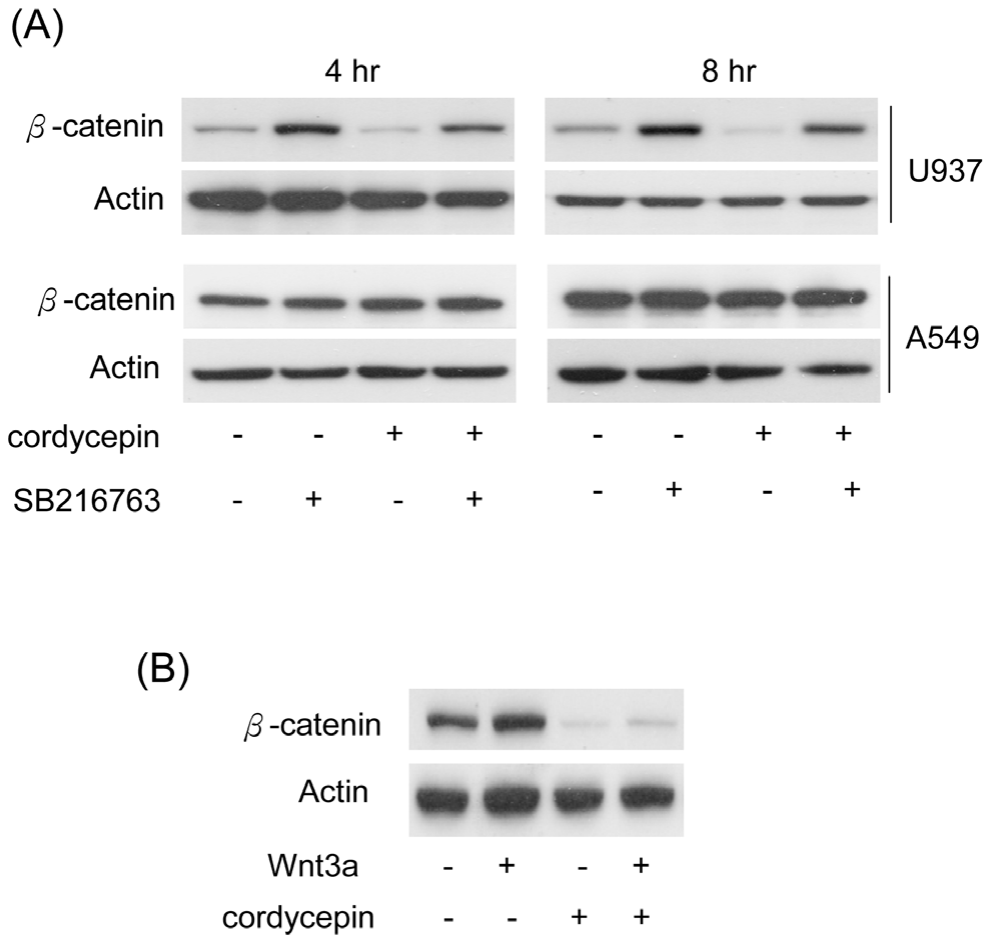
Cordycepin-suppressed β-catenin is mediated by Wnt and GSK3β-dependent pathways. (A) U937 and A549 cells were treated with cordycepin (100 µM) combined with/without a GSK-3β inhibitor (SB216763, 1 µM). Expression level of β-catenin was determined by Western blot analysis. (B) U937 cells were treated with/without Wnt3a (20 ng/ml) and cordycepin (100 µM), as indicated, for 4 hrs. Expression level of β-catenin was determined by Western blot analysis. Actin was used as loading control.

Stability of β-catenin is tightly controlled by GSK-3β. GSK-3β phosphorylates β-catenin and subsequently facilitates the ubiquitination and degradation of β-catenin. We assumed that cordycepin-induced β-catenin degradation is mediated by regulating GSK-3β. To investigate whether cordycepin influences GSK-3β activity, U937 and A549 cells were treated with cordycepin alone or in combination with SB216763, a pharmacological inhibitor of GSK-3β, for 4 or 8 hrs. Cordycepin-reduced β-catenin was significantly restored by SB216763. No such effect was found in A549 cells treated with either cordycepin alone or combined with the GSK-3β inhibitor (Figure 5A). In addition, treatment with Wnt3a induced β-catenin expression in U937 cells (Figure 5B lane 2 *vs.* lane 1). However, Wnt3a-induced β-catenin was significantly abolished by treatment of cordycepin (Figure 5B, lane 4 *vs.* lane 2). These results reveal that cordycepin suppresses expression of β-catenin in leukemia and this effect is mediated through modulating Wnt/GSK-3β signaling.

### PI3-K/Akt is Involved in Cordycepin-reduced β-catenin Expression

**Figure 6:**
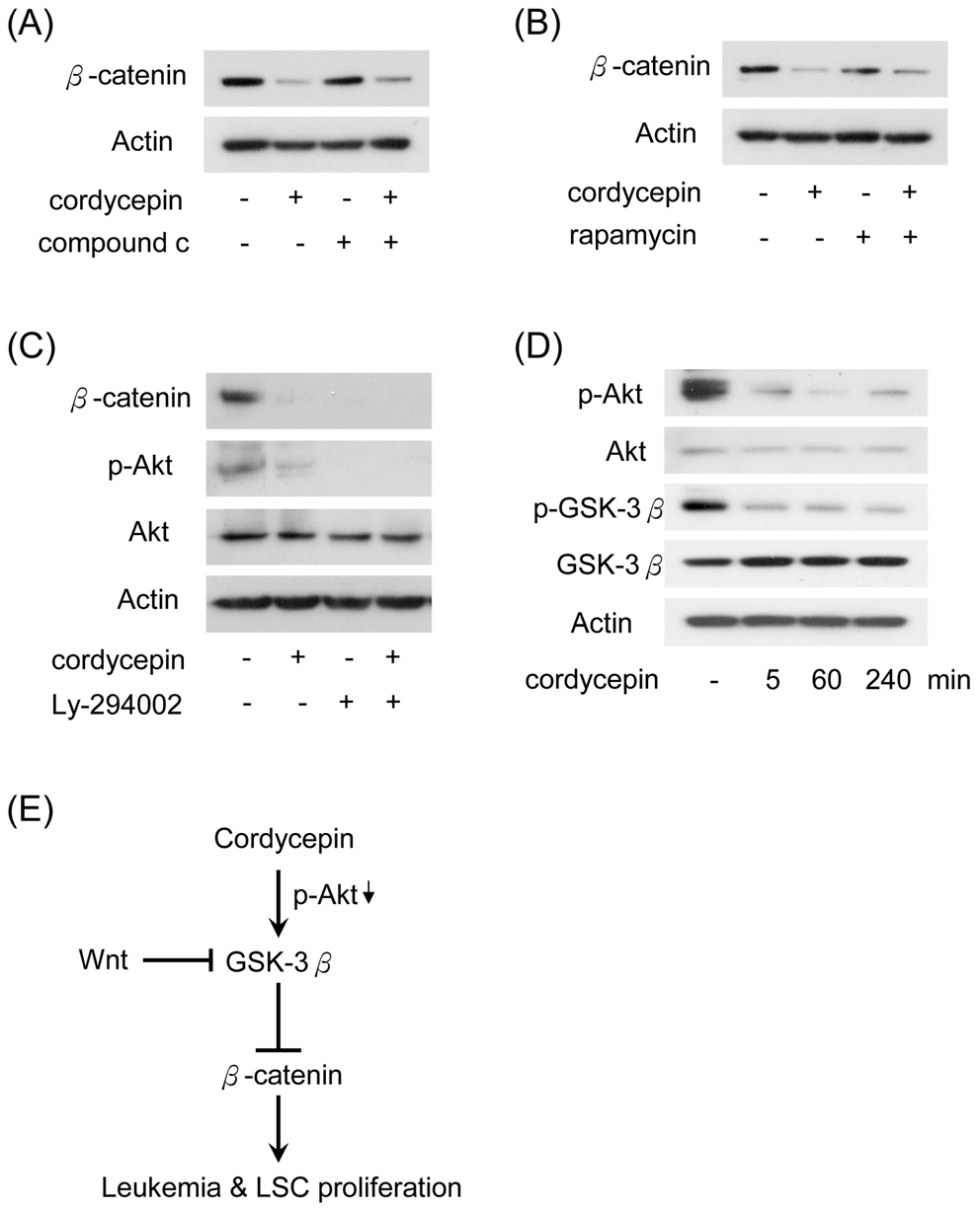
PI3-K/Akt signaling is involved in cordycepin-suppressed β-catenin. U937 cells were treated with cordycepin (100 µM) combined with/without (A) compound c (1 µM), (B) rapamycin (10 µM) and (C) Ly-294002 (20 µM) for 4 hrs. Expression level of β-catenin, Akt phosphorylated Akt (Ser473) were determined by Western blot analysis. Actin was used as loading control. (D) U937 cells were treated with cordycepin (100 µM) for 5, 60 and 240 min. Expression level of phosphorylated Akt (Ser473), Akt, phosphorylated GSK-3β (Ser9) and GSK-3β were determined by Western blot analysis. Actin was used as loading control. (E) A schematic model illustrating the role of cordycepin in suppressing β-catenin in leukemia cells.

Cordycepin was shown to regulate mTOR signaling pathway by regulating AMPK and Akt pathways in NIH3T3 cells [41]. As GSK-3β is one of the downstream kinases of Akt, we hypothesized that cordycepin-reduced β-catenin may be modulated *via* the inactivating of mTOR, AMPK or Akt. To test this hypothesis, U937 cells were treated with inhibitors of AMPK (compound c), mTOR (rapamycin) and PI3-K (Ly-294002), alone or in combination with cordycepin. Inhibition of AMPK or mTOR had no significant effect on the suppression of β-catenin (Figure 6A and 6B). In contrast, treatment with Ly-294002 or cordycepin suppressed β-catenin expression (Figure 6C). Moreover, cordycepin significantly inhibited phosphorylation of Akt (Ser473) which is correlated with the reduction of β-catenin (Figure 6C). In addition, cordycepin reduced phosphorylation of Akt and GSK-3β (Ser9) in a time course-dependent manner (Figure 6D). These results indicate that cordycepin may regulate GSK-3β by inactivating PI3-K/Akt signaling, thereby inducing protein degradation of β-catenin (Figure 6E).

## Discussion

Previous studies indicated that cordycepin induced leukemia apoptosis and suppressed cell proliferation [21]–[23]. However, the molecular mechanism of how cordycepin affects leukemia proliferation, LSC renewal and survival remains unclear. Our novel findings show that cordycepin suppresses leukemia proliferation and colony formation. Reduced proliferation is mediated by inducing protein degradation of β-catenin. Since Wnt/β-catenin signaling participates in maintaining cell survival and renewal of LSCs, we hypothesized that cordycepin can be used as a supplement or synergistic treatment for leukemia therapy. Interestingly, although β-catenin was shown to play a pivotal role in modulating distinct types of tumor progression, we found that cordycepin selectively impairs β-catenin’s stability in leukemia cells. In adherent epithelial cells, β-catenin binds to tight junction protein E-cadherin at the intra-cytoplasmic domain to maintain cell polarity and interaction with surrounding cells. Sequestration of β-catenin with E-cadherin results in preventing β-catenin from translocation and degradation. Thus, the distinct responses to cordycepin may occur as a result of different subcellular localization of β-catenin in adherent cancer cells and leukemia. These results reveal that there are different regulatory mechanisms involved in suppressing tumor progression by cordycepin in various malignancies. The reasons that cordycepin selectively targets β-catenin in leukemia but not in other solid tumors is currently under investigation.

Cordycepin is the structural agonist of adenosine. In this study, we found that treatment with adenosine did not affect β-catenin stability and leukemia cell proliferation (Figure S1 and Figure S2). However, a GSK-3β inhibitor significantly restored cordycepin-reduced β-catenin level (Figure 5A). Cordycepin was reported to inhibit protein synthesis and cell adhesion through modulating AMPK and mTOR-dependent pathway in fibroblasts [41]. However, results from treatments with compound c and rapamycin reveal that AMPK and mTOR pathways are not involved in cordycepin-reduced β-catenin (Figure 6A and 6B) in leukemia cells. In contrast, Ly-294002 dramatically reduced β-catenin level in leukemia cells (Figure 6C). Thus, cordycepin may regulate GSK-3β/β-catenin via a PI3-K/Akt dependent mechanism.

Increasing numbers of studies have focused on targeting β-catenin to suppress LSCs. A combination of inhibiting BCR-ABL activity and β-catenin signaling is considered to be a potential therapeutic strategy for CML patients [9], [13]. Thus, our findings that cordycepin selectively inhibits β-catenin in leukemia cells provides a novel leukemia therapy. Although the minimal concentration of cordycepin to significantly suppress leukemia cell proliferation *in vitro* is 50 uM, cordycepin abolished leukemia colony formation at a much lower concentrations (even at 5–10 uM, Figure 3). These results indicate that cordycepin is more effective at targeting LSCs but higher concentrations are required to suppress leukemia proliferation. Since LSCs are major source for the initiation and maintenance of leukemia, cordycepin is therefore most likely to target LSCs instead of suppressing leukemia cell proliferation. Thus, our findings imply that a lower dose of cordycepin may be sufficient when combined with other anti-leukemia drugs for suppressing survival or renewal of LSCs in *in vivo* animal model or human subject. In addition, since cordycepin is extracted from Dong Chong Xia Cao, which has been used as a food supplement for many generations, we expect the safety profile of cordycepin to be favorable with minimal side effects. An *in vivo* model of leukemia treatment combined with cordycepin is currently ongoing to validate this hypothesis. Taken together, our findings support the concept that synergism of cordycepin with other treatments is a potential strategy for leukemia therapy *via* suppressing leukemia cells and eradicating LSCs.

## Supporting Information

Figure S1 **Effects of adenosine on cell proliferation.** A549, U937 and K562 cells were treated with different concentrations of cordycepin and adenosine (50 to 200 µM) for 24 and 48 hrs. Cell proliferation was determined by an MTT assay. These data are from three independent experiments. Each bar denotes mean ± S.E.M.
(TIF)

Figure S2 **Effect of adenosine on β-catenin expression.** U937, K562 and A549 cells were treated with cordycepin (100 µM) or adenosine (50 to 200 µM) for 4 hrs and the expression of β-catenin was determined by Western blot analysis. Actin was used as a loading control.
(TIF)

Figure S3 **Efect of cordycepin on cyclin D expression.** A549 and U937 cells were treated with 100 µM cordycepin for 2 or 4 hrs. The expression of β-catenin and cyclin D1 was determined by Western blot analysis. Actin was used as a loading control.
(TIF)
