# Supplementary figures and images for: Cordycepin Regulates GSK-3β/β-Catenin Signaling in Human Leukemia Cells
Source: PLoS One. 2013 Sep 26;8(9):e76320. doi: 10.1371/journal.pone.0076320 (PMC3784440; doi:10.1371/journal.pone.0076320)

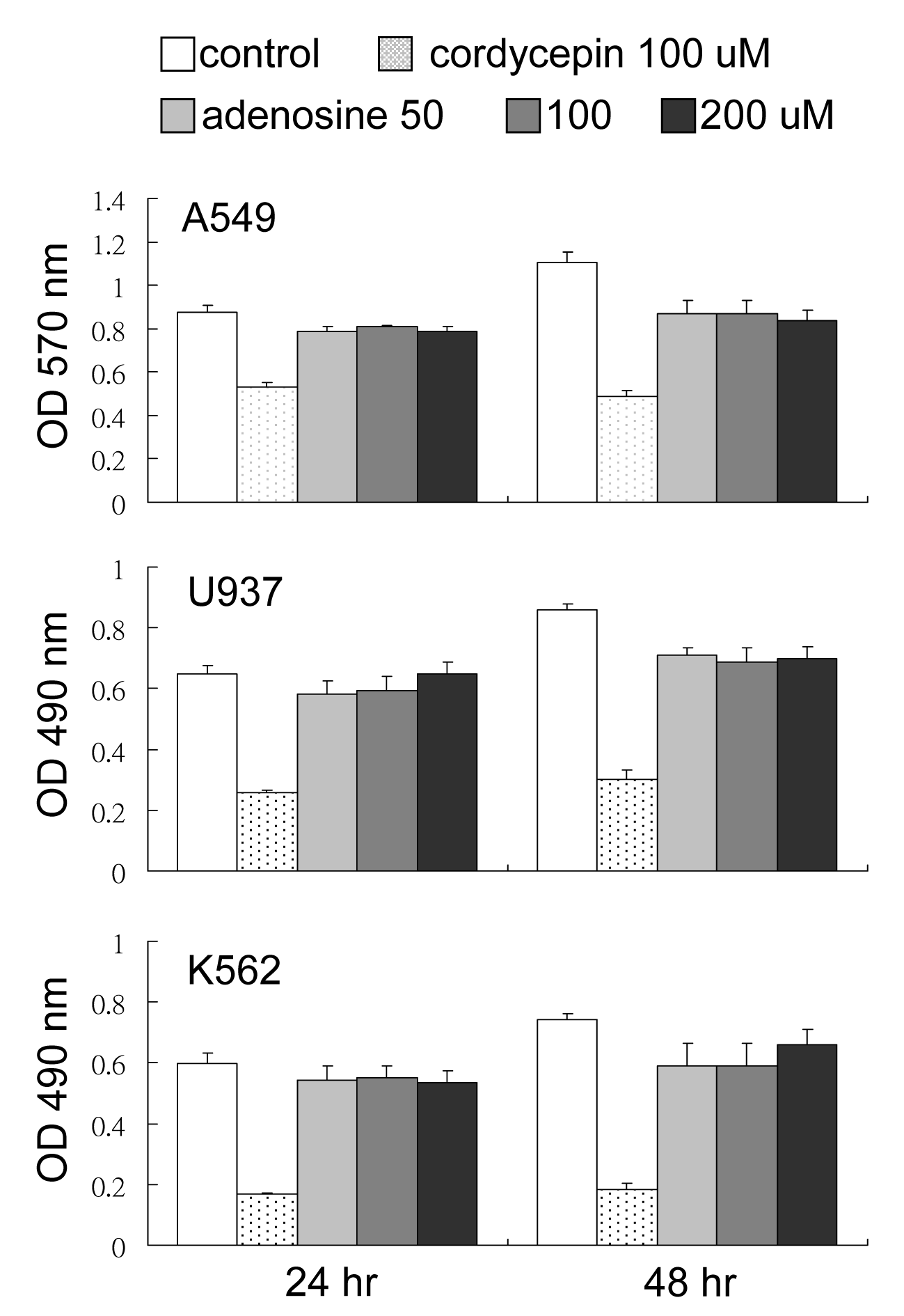

Supplement: Figure S1 — Effects of adenosine on cell proliferation. A549, U937 and K562 cells were treated with different concentrations of cordycepin and adenosine (50 to 200 µM) for 24 and 48 hrs. Cell proliferation was determined by an MTT assay. These data are from three independent experiments. Each bar denotes mean ± S.E.M. (TIF) [file pone.0076320.s001.tif]

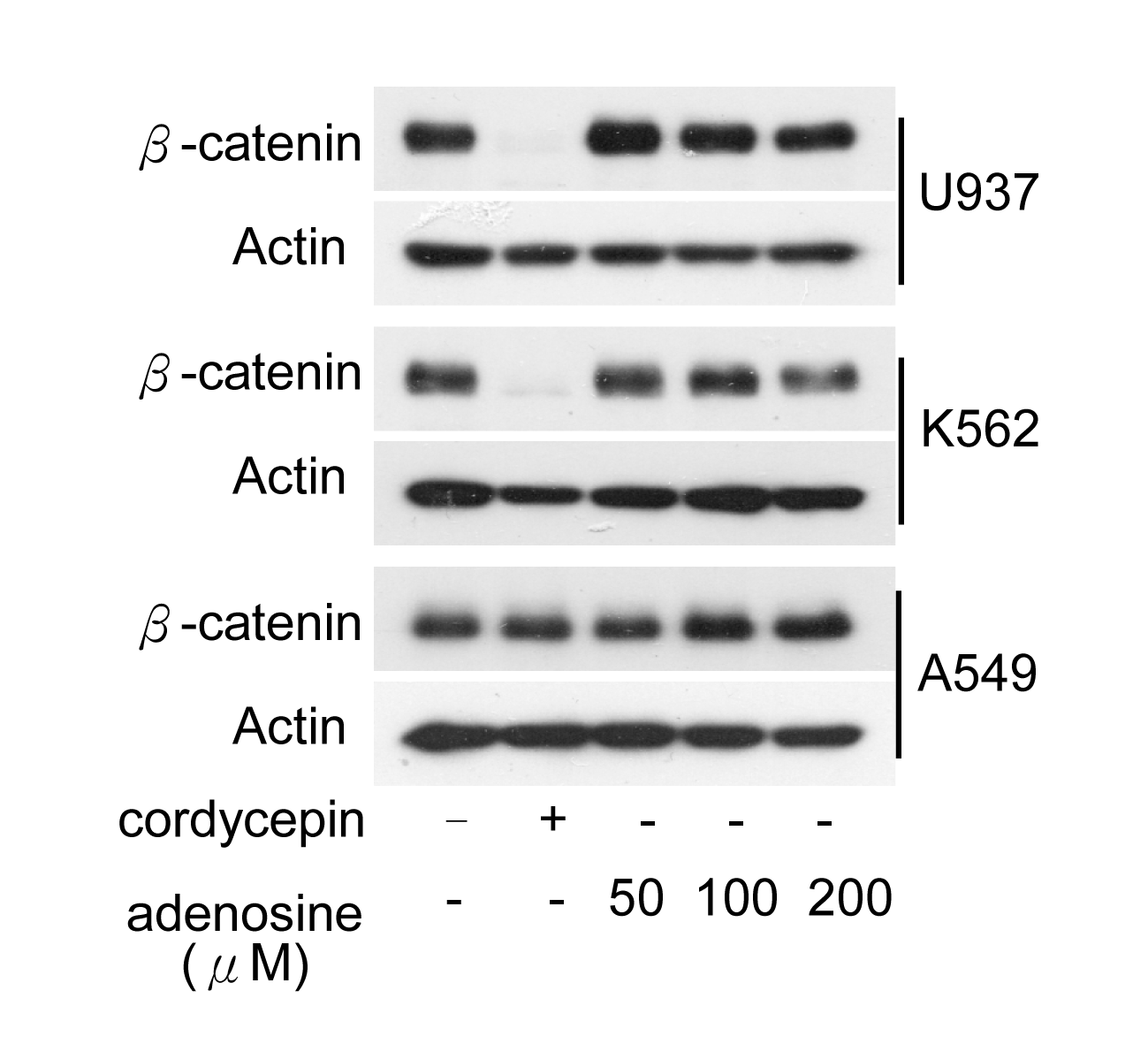

Supplement: Figure S2 — Effect of adenosine on β-catenin expression. U937, K562 and A549 cells were treated with cordycepin (100 µM) or adenosine (50 to 200 µM) for 4 hrs and the expression of β-catenin was determined by Western blot analysis. Actin was used as a loading control. (TIF) [file pone.0076320.s002.tif]

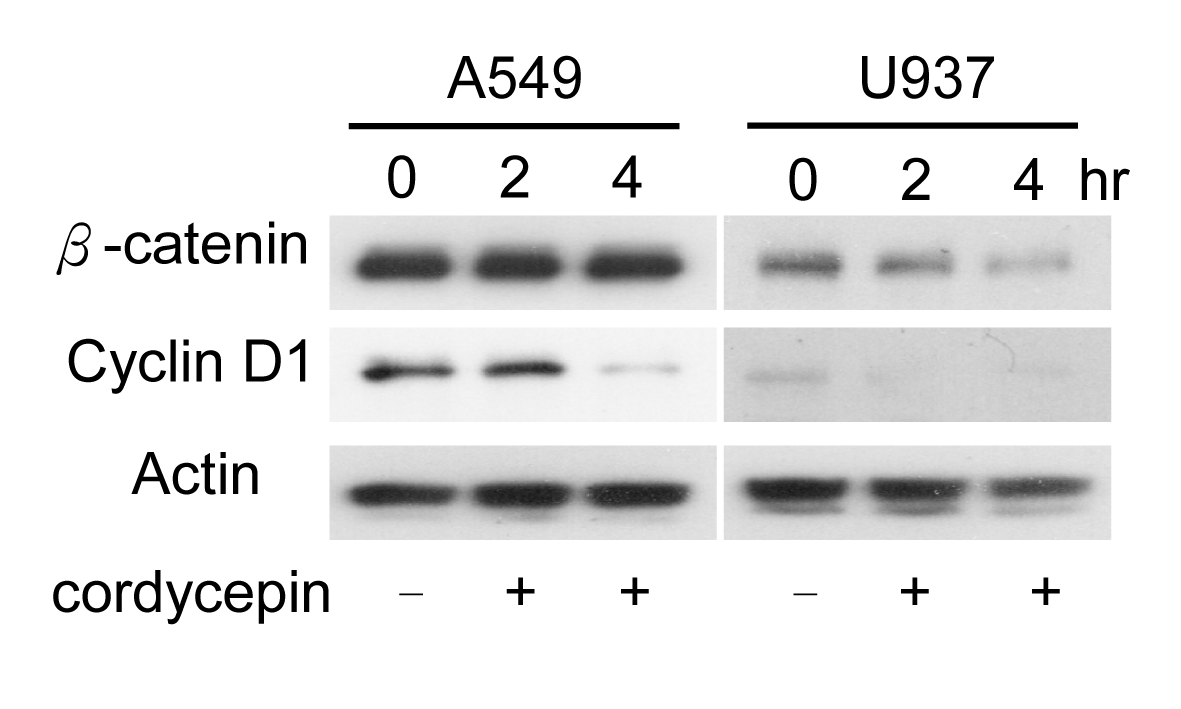

Supplement: Figure S3 — Efect of cordycepin on cyclin D expression. A549 and U937 cells were treated with 100 µM cordycepin for 2 or 4 hrs. The expression of β-catenin and cyclin D1 was determined by Western blot analysis. Actin was used as a loading control. (TIF) [file pone.0076320.s003.tif]
